# The Role of Self-Control and the Presence of Enactment Models on Sugar-Sweetened Beverage Consumption: A Pilot Study

**DOI:** 10.3389/fpsyg.2019.01511

**Published:** 2019-07-02

**Authors:** Mario Wenzel, Anouk Geelen, Maike Wolters, Antje Hebestreit, Kristof Van Laerhoven, Jeroen Lakerveld, Lene Frost Andersen, Pieter van’t Veer, Thomas Kubiak

**Affiliations:** ^1^Institute of Psychology, Johannes Gutenberg University Mainz, Mainz, Germany; ^2^Department of Agrotechnology and Food Sciences, Wageningen University & Research, Wageningen, Netherlands; ^3^Leibniz Institute for Prevention Research and Epidemiology – BIPS, University of Bremen, Bremen, Germany; ^4^Department of Electrical Engineering and Computer Science, University of Siegen, Siegen, Germany; ^5^Department of Epidemiology and Biostatistics, Amsterdam Public Health Research Institute, VU University Medical Center, Amsterdam, Netherlands; ^6^Division of Nutritional Epidemiology, University of Oslo, Oslo, Norway

**Keywords:** sugar-sweetened beverages, self-control, social norms, ecological momentary assessment, diet

## Abstract

The objective of the present research was to investigate associations of dispositional and momentary self-control and the presence of other individuals consuming SSBs with the consumption frequency of sugar-sweetened beverages (SSBs) in a multi-country pilot study. We conducted an Ambulatory Assessment in which 75 university students (52 females) from four study sites carried smartphones and received prompts six times a day in their everyday environments to capture information regarding momentary self-control and the presence of other individuals consuming SSBs. Multilevel models revealed a statistically significant negative association between dispositional self-control and SSB consumption. Moreover, having more self-control than usual was only beneficial in regard to lower SSB consumption frequency, when other individuals consuming SSBs were not present but not when they were present. The findings support the hypothesis that self-control is an important factor regarding SSB consumption. This early evidence highlights self-control as a candidate to design interventions to promote healthier drinking through improved self-control.

## Introduction

Sugar-sweetened beverage intake (SSB), including soft drinks, juices with added sugar as well as so-called sports and energy drinks, has been highlighted as one of the contributors to the increase in weight gain and lifestyle-associated diseases such as type 2 diabetes (World Health Organization [WHO], 2017). SSB consumption is frequent, with nearly 500 ml per day in the United States and contributes significantly to overweight and obesity given its poor nutritional value and high energy content accompanied by low satiation (Malik et al., 2006).

The abundant availability of SSBs as part of the obesogenic environment may facilitate consumption: Individuals are regularly confronted with a broad range of unhealthy food options which tempts indulging in those foods much more than exerting control over one’s own eating to achieve long-term health benefits (Stroebe et al., 2008). In fact, perceiving food in the environments activates neural responses in brain regions associated with gustatory and reward-related sensations which is particularly pronounced in individuals who report difficulties in weight regulation (Stice et al., 2008). Hence, individuals need to frequently exert self-control to protect their weight goals against those tempting food and beverages options, which offer immediate emotional satisfaction when consumed (Coumans et al., 2018a, b). The protection of the goal to control one’s own weight when confronted with the rewarding effects of sugar-rich food is effortful (Stroebe et al., 2013). Thus, it comes as no surprise that individuals who want to restrain their eating behavior often report difficulties shielding themselves from respective temptations (Wu et al., 2016). Recent research showed that individuals high in self-control reported less conflict involving foods and could resolve self-control conflicts faster than individuals low in self-control (Gillebaart et al., 2016). Moreover, it has been shown that self-control is especially important for restrained eaters, such that only restrained eaters with high levels of self-control tended to have a normal weight (Keller and Siegrist, 2014). Furthermore, there is ample evidence that executive functions such as working memory, which are closely related to self-control (Hofmann et al., 2012b), not only directly impact one’s eating behavior but also moderate the associations between food temptations and the actual eating behavior (Dohle et al., 2018). Finally, a systemic review indicated that food-related impulsivity was related to obesity and suggested that practicing inhibitory control provides a promising research avenue for weight loss or maintenance interventions (Giel et al., 2017).

Regarding SSBs, evidence on the role of self-control is scarce. One study demonstrated that the Theory of Planned Behavior can explain 38% of variance in self-reported SSB consumption over the past month (Zoellner et al., 2012). Perceived behavioral control (e.g., the perceived capacity to limit one’s own SSB consumption) showed a strong association of *r* = 0.54 with intention toward lower SSB consumption, which in turn predicted SSB consumption by facilitating implementation intentions (e.g., plans to limit one’s own SSB consumption). It is important to note that perceived behavioral control also capture perceptual aspects that are often not present in self-control conceptualizations. However, other research using more conventional measures of self-control demonstrated similar associations, such that poorer self-control performance in a Go/Nogo task was associated with higher SSB consumption (Ames et al., 2014). Thus, these results provide early evidence for the importance of self-control in SSB consumption.

The study by Zoellner et al. (2012) also demonstrated that social norms may be another important factor in contributing to SSB consumption, in that descriptive social norms provide information on how to act appropriately in certain situations. In the context of SSB consumption, this may lead to more difficulties for individuals to maintain a healthy diet since the more prominent examples of unhealthy diets may form unhealthy social norms (Christakis and Fowler, 2007), possibly encouraging individuals to eat less healthy in order to conform to the majority (Schultz et al., 2007). In fact, unhealthy descriptive norms in the form of present enactment models, that is other individuals who are actively engaging in the behavior (e.g., drinking SSB) that the participant aims at regulating (Hofmann et al., 2012a), have been found to be negatively associated with less healthy food choices (Mollen et al., 2013). Taken together, investigating influences regarding SSB intake stemming from self-control and descriptive social norms seems a fruitful avenue to gain better insight in behavior, and may provide entry points for behavior change approaches to prevent or reduce overweight and obesity.

The present research was part of the DEDIPAC (Determinants of Diet and Physical Activity) Knowledge Hub within the European Joint Programming Initiative Healthy Diet for a Healthy Life (JPI HDHL). Given the relatively scare evidence regarding the influence of self-control on SSB consumptions and its interplay with the presence of enactment models, we followed an ambulatory assessment approach, which often involves repeated measurements in the everyday life (Kubiak and Stone, 2012). Ambulatory assessment has many advantages which makes it valuable complementary tool to laboratory research on eating behavior. First, it allows for studying behavior within-person and between-persons which enables the researcher to go beyond the study of associations between obesity and food-related impulsivity and to investigate the trajectories of weight fluctuations and impulsivity within a person across time (Conner and Barrett, 2012). Second, it allows to capture situational influences more closely to when they happen and where they happen in the individual’s natural habitat. Third, participants are often asked to report momentary levels of variables of interest which reduces cognitive bias associated with self-reports (Trull and Ebner-Priemer, 2013), which is a frequent problem in research on eating and drinking (e.g., Westerterp and Goris, 2002). Building on these advantages, we conducted a pilot study using ambulatory assessment to investigate associations between *ad libitum* SSB consumption, measures of self-control, and the momentary social environment. Due to the pilot character of the method, we recruited a convenience sample and focused on self-control, thereby deliberately leaving out other important factors such as dieting goals and restrained eating that are known to influence eating and drinking behavior (Lowe et al., 2013).

## Materials and Methods

### Participants

University students were recruited on campus in two waves at four different study sites: Bremen (Germany), Mainz (Germany), Oslo (Norway), and Wageningen (Netherlands). Participants were included if they were aged between 18 and 30 years, were fluent in the language of the study site, drank at least on glass (200 ml) of SSB per week, and possessed a compatible smartphone (Android 4.0+; 100 MB available). They were excluded if they were pregnant or breast feeding (self-report), students of nutrition, food, or sports science studies, self-reported a mental disorder, diabetes mellitus or other relevant disease affecting metabolism or had a BMI <18.5 or >35.0 (self-report). As a means of compensation, participants received a remuneration of approximately $50 (Wageningen), a gift card of about $25 (Oslo), course credits (Mainz), or no compensation (Bremen). A total of 83 participants were recruited: 15 participants in Bremen, 35 in Mainz, 14 in Oslo, and 19 in Wageningen. Data from eight participants were excluded due to low compliance with the protocol (signal compliance <33%), leaving a final sample of 75 participants (52 females, age *M* = 22.9 years, *SD* = 3.3) with an average BMI of 21.5 kg/m^2^ (*SD* = 2.6). The study protocol was approved (Bremen and Mainz) or received a positive advice (Wageningen) by the local ethics committees of the respective study site or was approved by the Data Protection Official for Research in Norway (Oslo). All participants provided informed consent.

### Design and Procedure

During a first laboratory session, participants completed a questionnaire on dispositional self-control. Next, the open-source Android application “MyHealthAssistant,” which presents questionnaires and stores their data, was installed onto the participants’ smartphones or was pre-installed on the smartphone that was handed out to the participant. Starting the next morning after waking up, participants received signal-triggered prompts to complete self-reports on state self-control and on the presence of enactment models. Six signals were randomly distributed within 14 h with two consecutive signals required to be at least 30 min apart (*M* = 100.6 min, *SD* = 37.2, range = 31.2 to 250.8). Additionally, beverage consumption was assessed using event sampling, in that participants were instructed to register each drink with an app on their smartphone. After 1 week, participants returned to the laboratory for a second session where they were debriefed and completed a questionnaire to judge compliance and reactivity.

### Measures

To assess dispositional self-control, participants completed the Brief Self-Control Scale (BSCS; Tangney et al., 2004) that was translated by the members of the respective study site, except for the German study sites which used the validated German adaptation of the BSCS (Bertrams and Dickhäuser, 2009). The BSCS consists of 13 items (1 = not at all to 5 = very much) with higher scores indicating higher levels of dispositional self-control (*M* = 3.29, *SD* = 0.50, Range: 2.23 to 4.15). The scale’s reliability in this study was good, with Cronbach’s α = 0.77.

We also assessed several state measures. Participants completed the State Self-Control Capacity Scale (SSCCS; Ciarocco et al., unpublished). The two study sites in Germany used the validated German adaptation of the SSCCS (Bertrams et al., 2011), whereas the Dutch and Norwegian versions were translated by the authors. The scale consists of 10 items measuring one’s capacity to regulate oneself in a given moment and ranges from 1 (not true) to 7 (very true), with higher scores indicating higher levels of momentary self-control (*M* = 5.14, *SD* = 0.78). The between-person reliability of the SSCCS was very good (*R*_KRN_ = 0.91; Shrout and Lane, 2012), whereas the within-person reliability for changes from time point to time point was acceptable (*R*_CN_ = 0.67).

To assess the presence of enactment models, participants were asked whether other people were present and whether other physically present people in their environment were drinking SSBs (presence of other individuals consuming SSBs).

To capture beverage consumption, participants were instructed to (a) select the beverage from a precompiled list on their smartphone whenever they consumed a beverage and (b) scan the barcode on the beverage product if available. The list covered the following options: (1) milk and milk-based drinks, (2) coffee or tea, (3) water, (4) alcoholic beverages, and (5) other drinks (e.g., cola, energy drinks). If the option “other drinks” was selected, participants could choose between four SSB options (sugar-sweetened carbonated drinks, sugar-sweetened non-carbonated drinks such as nectar, sport drinks, and energy drinks) and comparable drinks not containing added sugar (diet carbonated drinks, non-carbonated diet drinks, smoothies, fruit-, or vegetable juice without added sugar). Next, the time at which a drink was consumed and the participant’s location was stored and participants were asked whether they wanted to scan a barcode. If an SSB was selected, participants were finally asked to indicate how much they drank (in glasses that were defined as 200 ml). The mean lag between beverage consumption (event-based sampling) and the assessment of momentary self-control and presence of other individuals consuming SSBs (signal-contingent sampling) was approximately half an hour (*M* = 34.3 min, *SD* = 14.73, Range: 0 to 191.73).

### Statistical Analyses

To assess adherence with the study protocol, we first investigated the proportion of completed self-report observations to the total self-report observations. We conducted a three-level mixed logistic regression with signal as the outcome (1 = completed, 0 = not completed) where signals were nested within days nested within participants. We included signal and day as predictors in this model and controlled for study site and wave as well as for age and gender. For the main analyses, we computed multilevel models with random intercepts with observations (Level 1) nested within participants (Level 2). For SSB consumption frequency, the Odds ratio (OR) was estimated by logistic regression with robust error variance, such that we regressed SSB consumption (0 = SSB not consumed or other drink consumed, 1 = SSB consumed) on the predictors of interest. For SSB consumption amount in ml, estimates were derived from a mixed regression with robust error variance. In the first step of both models, we regressed the frequency or the amount of SSB consumption on the grand-mean centered dispositional self-control, the time lagged (*t–*1) person-mean centered momentary self-control and the lagged (*t–*1) presence of other individuals consuming SSBs, controlled for day of study, signal number, study site, wave, gender, age, and BMI. We used the lagged variables to capture the levels of momentary self-control and the presence of other individuals consuming SSBs before and not after the SSB consumption since SSB consumption was coded as consumption within the last and the current signal. In the final step, we included the two-way interactions between either dispositional and momentary self-control, and presence of other individuals consuming SSBs. Statistical significance was accepted at *p* < 0.05 (two-sided). Given the nature of a pilot study and scarce prior evidence, the sample size was determined by the feasibility of recruitment. The minimally detectable effect size for a statistical power of 80%, alpha level of 5%, a total sample size of 75 participants, a base rate of 25%, and a two-sided test was an odds ratio of 1.81 or 0.55. The data and analysis script are available at https://osf.io/3ydrf/?view_only=6a50c9007ead4925b5fd791a36e6b47d.

## Results

### Descriptive Statistics and Adherence

The descriptive statistics of all measures can be found in Table 1. The three-level mixed logistic regression revealed a good overall adherence to the questionnaires, with 74.1% completed signals. Participants registered a total of 166 SSBs, with participants reporting to consume on average 2.21 glasses of SSBs (*SD* = 2.44) per week. Participants reported to drink more frequent SSBs over the course of a day, *OR* = 1.15, *z* = 2.37, *p* = 0.018, but the frequency of SSB consumption did not change from day to day, χ^2^(6) = 4.41, *p* = 0.621. Moreover, adherence to the completion of signaled questionnaires decreased from day to day over the course of the study, *OR* = 1.18, *z* = 5.70, *p* < 0.001. Thus, we included signal and day as control variables in the main analyses.

**TABLE 1:** Means, Standard Deviations, Range, and Zero-order Correlations of aggregated variables.

	**M / Proportion**	**SD**	**Range**	**1**	**2**	**3**	**4**	**5**	**6**	**7**
(1) Age	22.85	3.25	18–30	–						
(2) Female	69.3%	–	–	–0.02	–					
(3) BMI	21.50	2.60	16.17–29.65	0.05	0.06	–				
Underweight (<18.5)	8.0%	–	–							
Normal weight (18.5–25)	82.7%	–	–							
Overweight (25–30)	9.3%	–	–							
Obese (>30)	0%	–	–							
(4) Dispositional SC	3.29	0.50	2.23–4.15	0.07	–0.12	–0.12	–			
(5) Momentary SC	5.14	0.78	3.14–6.84	0.10	–0.06	0.22	0.37^∗∗^	–		
(6) Presence of EM	0.35	0.19	0.07–0.92	–0.00	–0.04	0.13	0.08	0.08	–	
(7) Frequency of SSB consumption	0.04	0.05	0–0.32	–0.03	0.15	0.10	–0.10	0.10	0.17	–
(8) Amount of SSB consumption in ml	27.71	43.20	0–200	–0.06	0.18	0.07	–0.17	–0.11	0.30^*^	0.75^∗∗∗^

SC, self-control; EM, enactment models. ^*^p < 0.05, ^∗∗^*p* < 0.01, ^∗∗∗^*p* < 0.001.

### Self-Control, Presence of Other Individuals Consuming SSBs and SSB Consumption

As indicated in Table 2, the multilevel models on the frequency and amount of SSB consumption revealed that dispositional but not momentary self-control was significantly associated with SSB consumption in both the unadjusted and the adjusted analyses. This means that participants high in dispositional self-control (1 SD above the mean) reported nearly half as much episodes of SSB consumption in the adjusted analyses, *b* = 0.05, *SE* = 0.01, *p* < 0.001, 95% (0.03, 0.06), than participants low in dispositional self-control (1 SD below the mean), *b* = 0.03, *SE* = 0.01, *p* < 0.001, 95% (0.01, 0.04). This differences was even more pronounced for the amount of SSB consumption; such that participants high in dispositional self-control consumed nearly one third of the amount, *b* = 13.49 ml, *SE* = 5.81, *p* = 0.020, 95% (2.09, 24.89), compared to participants low in dispositional self-control, *b* = 37.87, *SE* = 6.19, *p* < 0.001, 95% (25.74, 50.00).

**TABLE 2:** Estimates of the multilevel models with SSB consumption (either frequency or amount) as the outcome and dispositional and momentary self-control as well as presence of enactment models and their two-way interactions as the predictors.

	**Frequency of SSB consumption**				**Amount of SSB consumption in ml**			
	**Unadjusted**		**Adjusted**		**Unadjusted**		**Adjusted**	
		
	**OR**	**CI_95_**	***b***	**CI_95_**	**OR**	**CI_95_**	***b***	**CI_95_**
**Step 1**								
Dispositional SC	0.40^∗∗^	(0.22, 0.74)	0.52^*^	(0.29, 0.93)	–31.17^∗∗^	(−48.13, −14.21)	–24.69^∗∗^	(−42.04, −7.34)
Momentary SC^a^	0.82	(0.58, 1.17)	0.81	(0.57, 1.17)	7.22	(−16.20, 1.77)	–5.85	(−14.87, 3.16)
Presence of EM^a^	1.29	(0.79, 2.11)	1.28	(0.79, 2.09)	7.33	(−6.02, 20.68)	7.72	(−5.60, 21.04)
**Step 2**								
Dispositional SC × presence of EM^a^	0.53	(0.23, 1.23)	0.64	(0.30, 1.34)	–22.42	(−47.24, 2.40)	23.16	(−47.92, 1.60)
Momentary SC^a^ × presence of EM^a^	1.94^*^	(1.05, 3.59)	2.13^*^	(1.07, 4.23)	31.45^∗∗^	(12.55, 50.34)	29.99^∗∗^	(11.11, 48.87)

SC, self-control; EM, enactment models. ^*a*^Variable is time lagged (*t*–1). Adjusted for day of study, signal number, study site, wave, gender, age, and BMI.^*^p < 0.05, ^∗∗^*p* < 0.01, ^∗∗∗^*p* < 0.001.

When including the two-way interactions, the presence of other individuals consuming SSBs did not significantly moderate the association between dispositional self-control and SSB consumption. However, it did for momentary self-control in both the unadjusted and adjusted analyses, in that having more self-control than usual was only beneficial regard to lower SSB consumption frequency in the adjusted analyses, when other individuals consuming SSBs were not present, *b* = −0.02, *SE* = 0.01, *p* = 0.020, 95% (−0.03, <−0.01), but not when they were present, *b* = 0.01, *SE* = 0.01, *p* = 0.420, 95% (−0.02, 0.04). These results were mirrored by the results for the amount of SSB consumption, with *b* = −16.36 ml, *SE* = 5.71, *p* = 0.004, 95% (−27.55, −5.17) (not present) and *b* = 13.63 ml, *SE* = 7.68, *p* = 0.076, 95% (−1.42, 28.67) (present).

## Discussion

In this pilot study, we found that individuals with high levels of dispositional self-control reported lower SSBs consumption frequencies than individuals with low levels, demonstrated by half as much reported episodes of SSB consumption. This finding contributes to a growing body of evidence documenting the benefits of self-control for health-relevant behaviors (Ridder and Gillebaart, 2017). However, both momentary self-control and descriptive social norms represented by presence of enactment models (other individuals consuming SSBs) did not show significant associations with SSB consumption. Instead, the relationship between momentary self-control and SSB consumption was moderated by the presence of enactment models, such that reporting more momentary self-control than usual (within-subject process) was only associated with lower SSB consumption when enactment models were not present.

Thus, the relationship between self-control and social norms on SSB consumption were not straight forward but rather demonstrate the importance of differentiating between trait and momentary measures. In the case of SSB consumption, how participants view their self-control capabilities in general (trait self-control, e.g., “I am good at resisting temptation”) may explain SSB consumption better than how participants view their self-control capabilities in the ebb and flow of daily life. However, momentary self-control was more sensitive to situational factors such as presence of other individuals consuming SSBs, demonstrating that having more self-control than usual only helped when not in the company of others consuming SSBs. These results also highlight the limits of momentary self-control, in that momentary levels of self-control were only negatively associated with SSB consumption when other individuals were not present. However, when others were present, this association disappears, which could either reflect that descriptive norms prevail or that the dieting goal of restraining SSB consumption is not as salient in or does not apply to situations where other individuals drink SSBs. Future research should differentiate between these possibilities by assessing the fluctuations of the dieting goal across situations.

From an applied perspective, we believe that efforts to reduce SSB consumption should be multi-factorial, including targeting environments and individual processes. Besides limiting access and promoting alternatives to SSBs or on government policies such as taxes per drink and limiting advertising of SSBs (Scharf and DeBoer, 2016), our findings provide evidence suggesting that fostering both dispositional and momentary self-control may represent a complementary strategy to reduce SSB consumption on an individual level (Teixeira et al., 2015). Future research would profit from conducting interventions targeting long-term change in SSB consumption by employing strategies aimed at improving self-control in daily life (Houben et al., 2011).

Given that we did not screen for participants with dieting goals nor incorporated manipulations to activate them, it may be surprising that dispositional self-control was negatively associated with SSB consumption. At least in our sample, it seems that SSB consumption is in conflict with dieting goals that are readily activated in daily life and that can be better controlled by individuals high in dispositional self-control. However, our sample is based on young university students with relatively low BMI and, thus, may not be suited to generalize to the general population. Interventions targeting this specific population may, thus, not only focus on health education in the risks SSB consumption but could also benefit from incorporating inhibitory control training as a way of improving self-control in daily life.

A key methodological strength of the present study is the use of an event-based Ambulatory Assessment design where participants registered beverages *in situ*, which not only helps to overcome the biases associated with retrospective self-reports but also enables to study processes unfolding within individuals with high ecological validity. However, there are also a number of potential limitations to be considered. First, we used a correlational design that cannot provide causal evidence for the associations of self-control and social norms on SSB consumption. Second, we used lagged variables to explain SSB consumption between two signals. Thus, we are not certain whether other people who were reported to be present at time point 1 were present during the episode of SSB consumption between time points 1 and 2. Third, the number of reported drinks was quite low with slightly more than two glasses per week. This is not surprising as our pilot study relied on student samples given the well-established negative association between SSB consumption and socio-economic status and education (Han and Powell, 2013). Fourth, since it is impossible within this framework to assess the number of drinking episodes that were not reported, we cannot determine the compliance regarding beverages reporting. Fifth, given the different languages at the different study sites, we could not use validated versions of the self-control questionnaires since these were only validated in German and, thus, had to be translated to Dutch and Norwegian. Sixth, we did not assess current dieting goals of the participants or whether they routinely or momentarily restrain their eating behavior. Given that the negative associations between self-control and BMI was more pronounced in individuals with high levels of restrained eating (Keller and Siegrist, 2014), considering and assessing dispositional as well as momentary levels in restrained eating could increase the effect sizes we found in our study and would allow to investigate the interplay between self-control and restrained eating. Seventh, our measure of assessing the presence of other individuals drinking SSBs cannot differentiate between the influence via descriptive norms, i.e., the perception that consuming SSBs is widely accepted and not ostracized by other individuals, from other more direct influences such as an easier availability of SSBs (e.g., through offered SSBs from the other individual). Future research could assess these social influences in more detail in order to more elaborately study the role of descriptive norms on SSB consumption.

To conclude, our pilot study shows that self-control is an important factor in SSB consumption, which is potentially important for the design of interventions targeting obesity by promoting healthier drinking and eating. While our data cannot replace intervention studies, it suggests that fostering self-control in daily life may help lowering SSB consumption. Future studies are needed to determine whether self-control can be improved by interventions, leading to benefits in terms of reduced SSB consumption and associated health outcomes.

## Data Availability

All datasets generated for this study are included in the manuscript and/or the supplementary files.

## Ethics Statement

This study was conducted according to the guidelines laid down in the Declaration of Helsinki and all procedures involving human subjects/patients were approved by the local ethics committees of the respective study site (Bremen and Mainz, GER) or by the Data Protection Official for Research in Norway (Oslo, NOR) or received a positive advice by the local ethics committee (Wageningen, Netherlands). Written informed consent was obtained from all subjects/patients. Verbal consent was witnessed and formally recorded.

## Author Contributions

All authors designed and performed the study. MW analyzed the data and wrote the manuscript with input in consultation with all authors.

## Conflict of Interest Statement

The authors declare that the research was conducted in the absence of any commercial or financial relationships that could be construed as a potential conflict of interest.
